# Thyroid Hormone Function in the Rat Testis

**DOI:** 10.3389/fendo.2014.00188

**Published:** 2014-11-05

**Authors:** Ying Gao, Will M. Lee, C. Yan Cheng

**Affiliations:** ^1^Center for Biomedical Research, Population Council, New York, NY, USA; ^2^School of Biological Sciences, The University of Hong Kong, Hong Kong, China

**Keywords:** testis, thyroid hormones, spermatogenesis, gap junction, blood–testis barrier, seminiferous epithelial cycle

## Abstract

Thyroid hormones are emerging regulators of testicular function since Sertoli, germ, and Leydig cells are found to express thyroid hormone receptors (TRs). These testicular cells also express deiodinases, which are capable of converting the pro-hormone T4 to the active thyroid hormone T3, or inactivating T3 or T4 to a non-biologically active form. Furthermore, thyroid hormone transporters are also found in the testis. Thus, the testis is equipped with the transporters and the enzymes necessary to maintain the optimal level of thyroid hormone in the seminiferous epithelium, as well as the specific TRs to execute thyroid hormone action in response to different stages of the epithelial cycle of spermatogenesis. Studies using genetic models and/or goitrogens (e.g., propylthiouracil) have illustrated a tight physiological relationship between thyroid hormone and testicular function, in particular, Sertoli cell differentiation status, mitotic activity, gap junction function, and blood–testis barrier assembly. These findings are briefly summarized and discussed herein.

## Introduction

Thyroid hormones play a crucial role in regulating development, differentiation, and metabolism in multiple mammalian tissues. Testis was regarded as a thyroid hormone unresponsive organ for many years. In the past two decades, however, mounting evidence has emerged demonstrating the presence of functional thyroid hormone receptors (TRs) in the testis (1, 2). These findings illustrate that thyroid hormones likely play an important role in testis function. Studies have demonstrated that thyroid hormones most notably T3 (3,5,3′-tri-iodothyronine) regulates Sertoli cell proliferation and differentiation during testis development including the assembly of the blood–testis barrier (BTB) (3–5). Moreover, it also induces Leydig cell differentiation and stimulates steroidogenesis in the rat testis (6). Several idothyronine deiodinases and thyroid hormone transporters have been identified in the testis (7–10), illustrating that these enzymes and transporters necessary to maintain the homeostasis of thyroid hormone are present in the testis. It is generally accepted that thyroid hormone acts as an important regulator in testis development. However, few studies focused on the role of thyroid hormone in regulating spermatogenesis in adult testis. Studies in recent years have suggested that altered thyroid status in adult males is associated with abnormal spermatogenesis, reducing sexual activity and impeding fertility (11–15), illustrating the crucial relationship between thyroid hormones and maturation status of Sertoli cells. In fact, TRα1 is a reliable marker of Sertoli cell maturation because its expression is considerably down-regulated in adult testes, and continual expression of TRα1 illustrates delayed Sertoli cell maturation in adult mice (16, 17). There are also reports in recent years that thyroid hormone is crucial to maintain gap junction (GJ) and BTB function, as well as BTB maturation during postnatal development. Our goal in this mini-review is to focus on the role of thyroid hormone and junction dynamics, in particular, the BTB function during spermatogenesis, providing an update on the current status of research in this area. We also highlight research areas that deserve attention in future studies. We first provide a brief outline regarding the role of thyroid hormone in testis development and testicular function since this information is closely related to the emerging field in which thyroid is a major player in junction dynamics during spermatogenesis.

## Thyroid Hormone Action

Thyroxin (3, 5, 3′, 5′-tetraiodothyronine, T4) is the major form of thyroid hormones released by the thyroid gland into the systemic circulation. Thyroxin, however, is a pro-hormone, which must be converted to tri-iodothyronine (3, 5, 3′-tri-iodothyronine, T3), which takes place primarily in the liver and kidney. T3 is the bioactive form of thyroid hormone that has high affinity for nuclear TRs (18, 19). A small amount of T3 and reverse T3 (rT3), however, is also produced by the thyroid gland (20). T3 mediates its effects via genomic and also non-genomic pathways. For the classical genomic pathway, T3 mediates its effects by TRs. In the nucleus, TRs usually forms heterodimers with retinoid X receptor (RXR), and this complex further binds to thyroid response elements (TRE) in the promoter region of a target gene to regulate gene transcription (21). In addition, thyroid hormone also regulates the release of thyrotrophin-releasing hormone (TRH) by the hypothalamus and of thyroid-stimulating hormone (TSH) by the pituitary gland (21) to serve as a feedback loop in the hypothalamic–pituitary–thyroid axis to maintain the physiological level of thyroid hormone in the systemic circulation. In contrast to the genomic pathway, which has a relatively long response time, ranging from hours to days, non-genomic pathways have short latency and are not affected by transcription or translation inhibitors. Thyroid hormone binds to the binding elements such as integrin αvβ3 located at the plasma membrane or within a cell to exert its effects. These non-gemonic effects include the regulation of ion influxes, kinase signaling pathways, amino acid accumulation, extracellular nucleotide levels, and vimentin phosphorylation via non-receptor protein kinases downstream (10). While T4 is a pro-hormone, it can bind to TRs but with low affinity, and the T4 liganded-TR is less stable versus the T3-liganded-TR. Nonetheless, T4 serves as an agonist to TRs at appropriate concentration (22), which also depends on receptor isoform and the presence of cellular cofactors (e.g., thyroid hormone receptor-associated protein 220, TRAP200) (23). In addition to the genomic pathway, T4 also initiates rapid non-genomic response by binding to integrin αvβ3 in the plasma membrane, leading to an increase in cellular amino acid accumulation (24–26). Collectively, these findings illustrate T4 has a limited functional role in mammalian cells.

## Thyroid Hormone Receptors in Testicular Cells

Thyroid hormone receptors (TRs) are able to mediate the effects of thyroid hormone via classical genomic pathway via two genes, *THRA* (TRα) and *THRB* (TRβ). Alternative splicing gives rise to several TR isoforms: TRα1, α2, α3, and β1, β2, β3 (21). It is known that TRβ2 is restricted to the anterior pituitary and hypothalamus (27), and TRβ3 is highly expressed in liver, kidney, and lung (28). Although TRα2 and TRα3 mRNA are detected in Sertoli cells, these receptors do not have T3-binding capacity (2, 29–31). But they may exert dominant negative effects by binding to TRE to suppress gene transcription (32, 33). More important, TRα1 and TRβ1 are the functional TR isoforms by mediating T3 signaling, and also T4 but to a lesser extent. Both TRα1 and TRβ1 were shown to be expressed by Sertoli and germ cells throughout development in the rat testis (1). These two TR isoforms are abundantly expressed in neonatal Sertoli cells, suggesting that Sertoli cells might be the target cell type for T3 in the developing testis. A study using TRαKO or TRβKO mice has demonstrated that TRα1 is the crucial TR isoform, which mediates T3 effects in neonatal Sertoli cells (34). In fact, TRα serves as a reliable marker of Sertoli cell maturity. Persistent expression of TRα in adult testes is a reliable indicator of undifferentiated Sertoli cells, such as in neonatal mice (4, 35, 36) and in mice following deletion of A-kinase anchoring protein 9 (AKAP9) that impedes Sertoli cell differentiation (37). Recently, a transgenic model in which mice expressed a dominant negative TRα1 only in Sertoli cells was generated. By using TRα^AMI^-SC mice, T3 was shown to be a potent regulator to arrest Sertoli cell mitotic proliferation, which is mediated by an activation of TRα1 via the Cdk4/JunD/c-myc pathway (38). This finding is consistent with earlier reports that neonatal hypothyroidism induced in mice or rats by neonatal treatment with a goitrogen leads to an increase in Sertoli cell number and daily sperm production, concomitant with an increase in testis weight, due to a failure of Sertoli cell differentiation, making them mitotically active (4, 36, 39, 40). Also, in rodents when Sertoli cells cease to divide at age ~15- to 17-day postpartum (dpp) to become fully differentiated, this event coincides with a surge in T3 that peaks in the systemic circulation (41), illustrating a reciprocal relationship between T3 and Sertoli cell mitotic activity and differentiation status. Collectively, these findings illustrate T3 is a regulator of Sertoli cell mitotic function and differentiation status in the testis. Furthermore, TRs are detected in germ cells by immunohistochemistry (1). For instance, TRα1 is expressed by spermatogenic cells from intermediate spermatogonia to mid-cycle pachytene spermatocytes (1), suggesting that T3 may also play a role in germ cell meiotic development. Additionally, TRs are also expressed by Leydig cells in the interstitial compartment of immature testes (1). In fact, it was reported that Leydig cell differentiation and steroidogenesis in postnatal rat testes were affected by T3 (42).

## Iodothyronine Deiodinases in Testis

**Figure 1:**
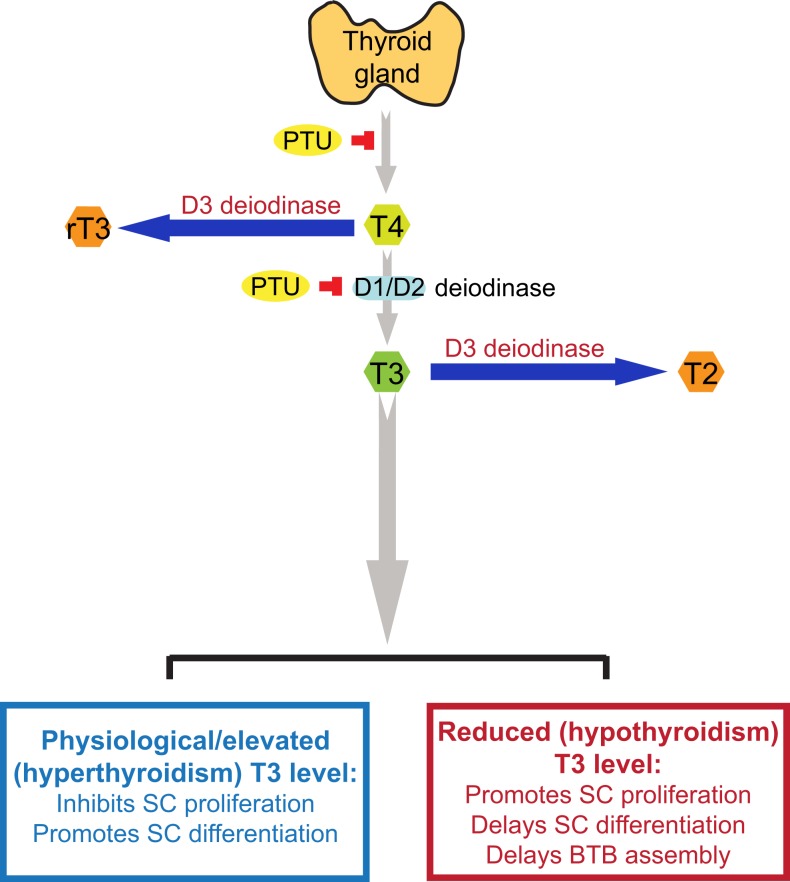
**A schematic drawing to illustrate the physiological role of thyroid hormone T3 on testis function**. This schematic drawing was prepared based on findings in the literature as discussed herein (see text for details). In short, T4 is the principal thyroid hormone produced by the thyroid gland and released into the systemic circulation. T4, however, is a pro-hormone, which is being activated via the action of deiodinases D1 or D2, mostly in the liver and kidney but also the testis, to form T3, the activated thyroid hormone. However, D1, D2, and D3 deiodinases are also found in the testis. The use of goitrogen [e.g., propylthiouracil (PTU)] can block the production of T4 by thyroid gland, which was used to examine the effects of thyroid hormones on testicular function. D3 deiodinase, unlike D1 and D2 deiodinases that activates T4 to T3, de-activates T4 or T3 to rT3 or T2, respectively, which are inactivated thyroid hormones, providing a crucial mechanism to regulate intracellular thyroid hormone action in cells, such as in Sertoli and/or germ cells in the testis. It is known that high level of T3 inhibits Sertoli cell proliferation and promotes Sertoli cell differentiation, whereas low level of T3 causes delayed Sertoli cell proliferation and differentiation. It is noted that at puberty (∼12 years of age) in men or by day ∼15-17 day in rodents, there is a surge in T3 level in systemic circulation, coinciding with Sertoli cell differentiation when Sertoli cells cease to divide (see text for details). T4, 3,5,3′,5′-tetraiodothyronine; T3, 3,5,3′-tri-iodothyronine; rT3, 3,3′, 5′-tri-iodothyronine; T2, 3,3′-diiodothyronine; PTU, propylthiouracil; SC, Sertoli cell; BTB, blood-testis barrier.

T4 released by the thyroid gland is the pro-hormone, which is converted to bioactive T3 by deiodination of T4 catalyzed by type 1 and type 2 deiodinase (D1 and D2; deiodinase is also known as iodide peroxidase), usually takes place in the liver and kidney (43) (Figure 1). Both the active hormone T3 and pro-hormone T4, however, can also be inactivated via deiodination by type 3 deiodinase (D3), converting into biologically inactive metabolites 3,3′-diiodothyronine (T2) and 3,3′,5′-tri-iodothyronine (reverse T3 or rT3) (43, 44), respectively (Figure 1). Thus, unlike D1 and D2 that activates thyroid hormones, D3 is an inactivator of thyroid hormones, serving as a modulator of intracellular thyroid hormone levels and action. All three deiodinases are detected in developing and adult testes (45). In developing testis, D3 is the predominant deiodinase and then its activity declines in adult testes (45), whereas D2 is the predominant activating deiodinase in the testis (42). D2 is abundantly expressed in elongated spermatids, whereas its expression could not be detected in Sertoli cells or other germ cells, suggesting that thyroid hormones might play a role in regulating spermatogenesis, specifically on spermiogenesis (9). However, the precise cellular localizations of D1 and D3 in the testis remain unclear. Earlier study has demonstrated that severe hypothyroidism may affect fertility in both sexes (46). Unexpectedly, mice lack either D1, D2 or both D1 and D2 are fertile and display normal serum T3 level (47–49). These findings indicate that in mice, D1 or D2 is not indispensable for maintaining serum T3 level, and D1 or D2-mediated local production of T3 is not likely to be the only source of T3 in the testis. Interestingly, knockout (KO) of D3 cause impaired fertility in mice, suggesting that D3 may play a more important physiological role in the testis (50). Thus, further studies are necessary to investigate the precise role of deiodinases in the testis.

## Thyroid Hormone Transporters in Testicular Cells

Since TRs and deiodinases are located intracellularly in mammalian tissues including the seminiferous epithelium in testes, thyroid hormones have to be transported across cell membranes before they can be activated by deiodinases, such as from T4 to T3, to mediate the effects via TRs or be inactivated, such as from T3 to T2 or T4 to rT3. While there is no specific membrane bound TRs, several membrane bound drug transporters are putative transporters of thyroid hormones that include monocarboxylate transporter (MCT) 8, MCT10, and organic anion-transporting polypeptides (OATPs) (51, 52). MCT8 is a specific thyroid hormone transporter. Unlike MCT8, MCT10 not only transports thyroid hormones but also aromatic amino acid. Both of MCTs prefer T3 over T4, and MCT10 is even more efficient than MCT8 in transporting T3 across plasma membranes (53). However, studies have shown that MCT8 KO, MCT10 KO, and MCT8/MCT10 double KO mice are all fertile in both sexes, supporting the notion that other thyroid hormone transporters may compensate the loss of MCT8 and MCT10 (54). Additionally, OATPs are able to transport steroid conjugates, prostaglandins, bile acids, drugs, and thyroid hormones (55). Several OATP family members have been detected in the testis (56). For instance, OATP-F, a homolog of OATP1C1, displaying high affinity for T4 and rT3, has been detected in human Leydig cells (57). OATP6A1, originally identified as a cancer/testis antigen also called SLCO6A1, is predominantly expressed in normal testes (58). In addition, two spliced variants of OATP3A1 called OATP3A1-V1 and OATP3A1-V2 have also been detected in germ cells and Sertoli cells, respectively (59). The rat gonad-specific transporter (GST)-1 and GST-2, which are members of OATPs family are highly expressed in Sertoli cells, spermatogonia, and Leydig cells (60), which may also be involved in T3 and T4 transport across the plasma membrane. A recent study has demonstrated that MCT8 and OATP1C1 are crucial to maintain the thyroid hormone homeostasis in the mouse brain (61), and OATP14 is a high affinity transporter for T4 at the blood–brain barrier (62). Much research is needed to delineate the physiological role of OATPs and MCTs in regulating thyroid hormone transport across the BTB.

## Effects of Thyroid Hormones on Sertoli Cell Proliferation, Differentiation, and BTB Assembly

Propylthiouracil is a goitrogen that inhibits the enzyme thyroperoxidase by blocking the production of T4 from thyroglobulin in the thyroid, causing hypothyroidism. It also inhibits 5′-deiodinase that converts T4 to T3. Thus, PTU is a widely used thiouracil-derived drug used to treat hyperthyroidism (63, 64). PTU-induced neonatal hypothyroidism by treating neonatal rats from birth was shown to increase rat testis weight and daily sperm production of up to 80 and 140%, respectively (35, 36). Further studies demonstrated that this was the result of Sertoli cell proliferation and a delay of Sertoli cell maturation (5). Furthermore, the Sertoli cell BTB failed to assemble by 15–25 dpp even though some tight junction (TJ) structures were detected by electron microscopy at these ages, but extensive network of TJ ultrastructure and basal ectoplasmic specialization (ES) analogous to age-matched control rats was not found in these rats treated with PTU from birth to age 25 dpp (5). Conversely, neonatal hyperthyroidism was found to stimulate Sertoli cell differentiation, rendering Sertoli cells ceased to proliferate by age 12 versus ∼15–17 dpp in normal rats, thereby reducing the testis weight in adult animals at age 100 dpp by almost 50% (3). These findings suggest that thyroid hormone regulates testis development by modulating Sertoli cells mitotic activity, differentiation status, and the BTB assembly. Table 1 summarizes some of the known effects of thyroid hormone T3 on Sertoli and Leydig cell function in the testis.

**Table 1:** **Effect of thyroid hormone T3 on testes**.

Cell type	Effects: stimulation (+), inhibition (−)	Reference
Sertoli cell	Proliferation (−)	(3, 4)
	Differentiation (+)	(3, 4, 39)
	ABP production (−)	(65)
	AR (+)	(30)
	Aromatase (−)	(66, 67)
	Connexin 43 (+)	(68)
	ER (−)	(69)
	GLUT1 (+)	(70)
	IGF-1 (+)	(71)
	Inhibin (+)	(3)
	Lactate (+)	(39)
	NCAM (−)	(72)
	Nidogen (+)	(73)
	p21^Cip1^ (+)	(74, 75)
	p27^Kip1^ (+)	(74, 75)
	Testosterone metabolism aromatization (−)	(39)
	Type IV collagen (−)	(73)
	Vimentin phosphorylation (+)	(76)
Leydig cell	Differentiation (+)	(77, 78)
	Steroidogenesis (+)	(79)
	StAR protein (+)	(79–81)

*ABP, androgen binding protein; AR, androgen receptor; ER, estrogen receptor; GLUT1, glucose transporter-1; IGF-1, insulin-like growth factor-1; NCAM, neural cell adhesion molecule*.

## Thyroid Hormones, Gap Junction, and Epithelial/Endothelial Barrier Function

Gap junctions are intercellular channels, which mediate direct communication between neighboring cells. These channels allow passage of ions and small molecules, usually <1–1.5 kDa, and are involved in several physiological processes, such as cell growth, apoptosis, and differentiation (82–85). Connexin 43 (Cx43) is the predominant GJ protein in the testis (84, 86), it is expressed by Sertoli cells, germ cells, as well as Leydig cells in the testis and found at the Sertoli cell–cell and Sertoli–germ cell interface (87, 88). Although the Cx43 germ line KO mice died shortly after birth due to heart defects, deletion of Cx43 was shown to induce germ cell deficiency in the testis of developing embryo (89). Interestingly, Sertoli cell-specific Cx43 KO (SC-Cx43 KO) mice have smaller testes, and the seminiferous tubules of these KO mice contain mitotically active Sertoli cells and early spermatogonia but not any other germ cell types since spermatogonia failed to differentiate into spermatocytes beyond type A to enter meiosis (90). It is noteworthy that Sertoli cells of SC-Cx43 KO mice remained proliferative in adult mutant mice (16, 90), analogous to the phenotypes of Sertoli cells in the goitrogen-induced hypothyroidism model. These findings also illustrate that Sertoli cell maturation is perturbed following deletion of Cx43 in these mutant mice. TRα1 mRNA expression was also found to be up-regulated by 20- and 60-dpp in the testis of SC-Cx43 KO mice versus the age-matched control (16). It is noted that TRα1 is abundantly expressed in the testis during neonatal period but rapidly declines in adulthood in normal rats (21). These findings thus illustrate an inactivation/deletion of Cx43 causes an upregulation of TRα1, which may mediate thyroid hormone action on Sertoli cell differentiation. Taken collectively, these data thus demonstrate unequivocally that Cx43 plays a crucial role in spermatogenesis and testis development, which is also involved in thyroid hormone action in the testis. In fact, studies have shown that thyroid hormone may inhibit Sertoli cell proliferation by up-regulating Cx43 expression (68, 91). However, the precise mechanism remains unknown. In tumor cells, overexpression of Cx43 induces cyclin-dependent kinase inhibitor (CDKI) p27^Kip1^ level (92). Consistent with this finding, *in vitro* studies have shown that T3 up-regulates p27^Kip1^ and p21^Cip1^, which, in turn, may play a role in down-regulating Sertoli cell proliferation (74, 75, 93). It is also likely that thyroid hormone regulates Cx43 expression, which in turn induces the expression of maturation/differentiation markers p27^Kip1^ and p21^Cip1^ via a yet-to-be defined signaling pathway, leading to an arrest of Sertoli cell proliferation. This possibility must be carefully evaluated in future studies to define the physiological relationship between Cx43 and thyroid hormone action in the testis as well as the involving signaling molecules.

While studies using goitrogen and Sertoli cell-specific Cx43 KO models have demonstrated the physiological relationship between thyroid hormone action, Cx43-based GJ function and spermatogenesis (e.g., differentiation of spermatogonia to spermatocytes and the onset of meiosis), in particular, the impact of T3 on Sertoli cell BTB assembly, the molecular mechanism(s) underlying these observations remain unknown. An early report has demonstrated that treatment of chick with thiouracil that inhibits T3 production also delays the development of interdigitation of the lateral plasma membrane between adjacent corneal endothelial cells whereas thyroxine treatment accelerates development of endothelial cell lateral borders (94). These findings are physiologically important to studies in the testis since Sertoli cell cytoplasmic processes create interdigital association with different germ cell types at a Sertoli:germ cell ratio of ∼1:30-1:50 during spermatogenesis, requiring extensive interactions between Sertoli and germ cells at the plasma membranes, supporting the notion that T3 may play a role in junction dynamics in the seminiferous epithelium. It is likely that T3-mediated Cx43-based GJ function may be crucial to these events. It is logical to use the goitrogen-induced hypothyroidism model in both neonatal and adult rats to examine changes in junction dynamics at the BTB and also Sertoli–germ cell interface during spermatogenesis in future studies.

## Concluding Remarks and Future Perspectives

Herein, we provide an update on the role of T3 on Sertoli cell maturation, differentiation and BTB assembly during development. Figure 1 summarizes the latest findings regarding the role of thyroid hormones in Sertoli cell proliferation, differentiation, and BTB assembly based on several reports in the last two decades investigating the role of thyroid hormones on testis function. However, there is a lack of data regarding the mechanism(s) by which T3 affects BTB developing at ~15- to 21-dpp in rats. Does this involve changes in the spatiotemporal expression, localization, and/or intrinsic activity of actin regulatory proteins, such as Arp2/3 (actin-related protein 2/3) complex (a branched actin polymerization inducing protein), palladin (an actin bundling/cross-linking protein), Eps8 (epidermal growth factor receptor pathway substrate 8, an actin barbed end capping, and bundling protein), which affect organization of actin microfilaments at the BTB? Does this involve changes in the endocytic vesicle-mediated protein trafficking, thereby impeding localization of adhesion protein complexes at the Sertoli cell–cell interface? What is the effect on the actin microfilament organization at the ectoplasmic specialization following knockdown of D1, D2, and/or D3 in Sertoli cells? Many of these questions will need to be addressed before we can gain some insightful information on the role of thyroid hormone on junction dynamics in the testis. Furthermore, selenium, a key element to maintain spermatogenesis and male fertility (95), is the prosthetic group of deiodinases, as such selenocysteine that plays an important role in determining the free circulating level of T3 in the mammalian body. As such, the involvement of selenium in thyroid hormone action should also be considered in future studies.

## Conflict of Interest Statement

The authors declare that the research was conducted in the absence of any commercial or financial relationships that could be construed as a potential conflict of interest.
